# Validation of a questionnaire exploring patient attitudes towards bedside teaching

**DOI:** 10.1186/s12909-022-03192-2

**Published:** 2022-03-07

**Authors:** MO Carey, N O’Riordan, M Carty, M Ivers, LK Taylor, MF Higgins

**Affiliations:** 1 Obstetrics and Gynaecology, National Maternity Hospital Dublin, Dublin, Ireland; 2 UCD School of Psychology, University College Dublin, Dublin, Ireland; 3 School of Psychology, Queen’s University Belfast, Belfast, UK; 4 Obstetrics and Gynaecology, UCD Perinatal Research Centre, National Maternity Hospital Dublin, 65-66 Lower Mount Street, D02YH21 Dublin, Republic of Ireland

**Keywords:** Questionnaire, Bedside Teaching, Patients

## Abstract

**Background:**

Bedside teaching (BST) facilitates medical education and has reduced in practice, often due to patient-related concerns. This study aimed to validate a questionnaire exploring patients attitudes towards BST.

**Methods:**

International guidelines for questionnaire development were followed. Seven steps were included: literature review, patient interviews, development of clear and understandable items, expert validation, cognitive interviewing and pilot testing. Statistical analyses included exploratory factor analysis, internal consistency, investigation of demographic influences and discriminant validity across subscales.

**Results:**

Following the literature review, 32 interviews were conducted. Potential items were developed, reviewed and adapted. Experts in medical education and statistics reviewed the draft questionnaire. Fifteen patients consented to cognitive testing and 401 consenting patients completed the final version. The median age of participants was 35 years of age (range: 18 to 70 years). Participants included women attending for antenatal (40%), postnatal (32%) and gynaecology issues (28%). Just under one third (29%) had taken part in medical student teaching previously.

Statistical analyses found a two-factor solution, consisting of *Educate medical professionals* and *Conditions for participation* subscales with good internal consistency; responses did not vary by age or education. Participants who had opted-in for teaching in the ward and bedside endorsed higher levels of *Educate medical professionals,* suggesting discriminant validity.

A majority of patients (> 92%) reported that they were happy to be involved in BST. Patients believed that they should not be asked to participate in BST should they feel stressed or unwell (68.2%).

**Conclusion:**

This study shows extensive patient support for BST, independent of age or education. The desire to educate is a strong motivating factor. This strong support by patients for BST is an area that medical schools and universities can potentially develop. Future versions of this questionnaire may include virtual bedside teaching, in the context of social distancing.

## Background

Bedside teaching (BST) involves clinical teaching in the presence of the patient, usually with a three-way relationship between clinical teacher, student and the patient [1]. BST is considered essential to help students develop their understanding of the doctor-patient relationship (particularly of patient-centred care), and to facilitate the development of students clinical reasoning, clinical and communication skills and professionalism [2–4]. The student, whether a medical student or graduate trainee in the speciality, experienced not just how to assess disease, but how to personally and professionally address the human impact of illness that perhaps cannot be conveyed by an actor [1–3, 5, 6]. This holistic teaching is popular with students [7, 8], many of whom regard it as the most effective way of teaching clinical skills [9, 10], an opinion that echoes Osler’s original principle that “*have no teaching without a patient for a text, and the best teaching is that taught by the patient himself*” [11].

In some countries, BST is in decline, a situation that may only be exacerbated by the SARS-CoV2 pandemic [12–15]. Fifty years ago, three-quarters of clinical teaching was documented as at the bedside; just over ten years ago estimates suggested 17% of teaching was at the bedside, with most of this being the teaching of physical examination [16]. There are multiple factors that may have resulted in this [17–19] including busier clinical environments, increased patient throughput, potential availability of teachers [20, 21] and other alternatives to BST, including simulation and virtual learning. There also is a perception that bedside teaching, as it was formerly practiced, is intruding or demeaning to patients [18].

Despite this perception, many of the publications that have studied patient perceptions of BST have found favourable results, with patients reporting that they found BST enjoyable, improved their understanding of disease and increased their perception of both physicians and trainees [7, 17, 22–28]. Most of these studies used unvalidated questionnaires, or studied only those who had already completed BST. A recent qualitative study from our group showed that patients who had completed a BST session were highly supportive of it, given that students and clinicians behaved respectfully and maintained confidentiality. Clinical learning was prioritised by patients as an important part of medical education. Patients showed altruism and positivity towards BST [25]. Limitations of this study were that it only focused on people who had experienced BST, and the majority of participants were university educated, thus studying a limited group. With the intention to extend the original work to those who may decline BST, and to other patient groups, we aimed to develop and validate a questionnaire on BST.

## Methods

This study was performed in the National Maternity Hospital, Dublin, a tertiary level unit with more than 9,000 births per year. The hospital provides undergraduate and postgraduate teaching to the full multidisciplinary maternity team.

Two types of bedside teaching occur within this hospital, full details of which are published elsewhere [25]. In brief, the first type is a senior midwife led multidisciplinary team ward round occurring every morning and based on the principles of family centred care [23]. This ward round focuses on clinical care; education is a secondary outcome and is provided only by qualified doctors training in the speciality of Obstetrics and Gynaecology. The second type is a teaching ward round occurring two to three times a week during term time, focused on education of medical students, following standard principles of Choice, Consent and Confidentiality [29] and led by trained medical educators. While the second type of BST was the focus of the qualitative research study [25], both types are referenced in the development of this questionnaire.

The Association of Medical Education in Europe guideline was followed (AMEE) [30], which advises a seven stage process. In the first stage, we identified domains relevant to bedside teaching by performing a thorough literature review. We then conducted interviews with patients for the second stage. Semi-structured interviews performed as part of the qualitative research paper were included. Further interviews were performed to include those who had declined to participate in BST, endeavouring to be respectful of their wish to decline participation while still gently exploring their reasons for declining. In the third stage, we synthesised the literature review and interviews to help us to develop potential items in the fourth stage. We constructed questions from these domains to make sure the concept of the questionnaire made sense and that the language was understandable. We then developed the questionnaire further to ensure the questions adequately represented the construct of patient’s perspective to BST.

The fifth stage involved conducting expert validation, where we asked a panel of experts in medical education and medical statistics to examine the draft questionnaire. The panel were tasked with ensuring that the questions were relevant to the construct being measured and that key items or indicators had not been omitted. The sixth stage involved us performing cognitive testing which was performed on a sample in order to test understanding of items and allowed us to develop a version that could be piloted as part of the seventh stage (Table 1).

**Table 1:** Version Nine of Questionnaire Items on BST (Bedside Teaching) for pilot testing. Items with stars (*) were excluded after statistical analysis

1. Taking part in BST could be an enjoyable experience for me
2. It would give me satisfaction to feel that I could help in the education of future generations of healthcare professionals by participating in BST
3. Medical terms used during BST which I do not understand should be explained to me
4. If I felt stressed or was feeling unwell I should not be asked to participate in BST
5. By participating in BST I could possibly learn more about my condition than I had previously known*
6. Hearing details about my condition being discussed in BST may make me feel anxious*
7. I think it is important to check what the students would do with the information they hear about me during BST
8. Before participating in BST the students should get my verbal consent
9. Before participating in BST the students should get my written consent
10. I am happy to help teach midwives
11. I am happy to help teach junior doctors
12. I am happy to help teach medical students
13. It is important to me that the team remains professional throughout BST
14. I feel if asked to participation in BST I would be able to say no*
15. I feel proud that pregnant women are in a unique position to help to educate students about pregnancy during BST
16. I am happy to follow the advice of a midwife or doctor when it comes to making a decision for clinical care
17. Do you have anything to add that you think may be important for us to know?

Following statistical advice, we aimed administer the questionnaire to 400 participants in multiple and diverse areas of clinical care (antenatal, postnatal, inpatient, outpatient, gynaecology, clinic attendance).

Based on these data, we conducted an exploratory factor analysis (EFA) with the aim of identifying the underlying latent, or unobserved, constructs among patients. Following the EFA, our exploratory analyses examined the potential link from demographic variables (e.g., age, education) as well as potential discrimination validity across the subsamples (e.g., those who have participated, or not, in teaching in the clinic, ward or bedside). The aim of these analyses was to understand the variables which may influence the pattern of responses to the newly developed scale.

We obtained Ethics Committee approval from the National Maternity Hospital Ethics Committee.

## Results

Scale Development: Following a thorough literature review, we conducted interviews aiming to develop relevant items. As well as the women interviewed in the qualitative research study (*n* = 22) [25], a further ten women were interviewed for this specific study, who had declined to participate in BST. Three women were invited but declined both to participate in BST and to be interviewed. We identified key themes in those who had participated in BST, which were professional mannerisms, privacy and personal wellbeing, quality of service, clinical experience and leaning importance [25]. Those whom had declined did so because they felt overwhelmed, felt too unwell or shy, or were uncertain of what the function of BST of was.

In the third stage, we developed, reviewed and adapted potential items to ensure that concept of the questionnaire made sense and that the language was understandable. We selected a five-point response scale response ranging from 1 (“*strongly disagree*”), 2 (“*disagree*”), 3 *(“neutral*”), 4 (“*agree*”) to 5 *(“strongly agree*”) and NA (“*not applicable*”), with the potential to merge points if there was minimal difference on analysis.

The fifth stage involved conducting expert validation, where we asked a panel of experts in medical education (three experienced clinical educators, ranging in experience from 15 to 30 years of teaching) and medical statisticians (two experienced statisticians, ranging in experience from five to thirty years) examining the draft questionnaire. We introduced additional items and changed phrasing (e.g. from “*medical and midwifery students*” in one item to “*medical students*” or “*midwifery students*” in two separate items).

We performed cognitive testing upon a sample of 15 consenting women, nine from obstetrics and six women attending as gynaecology patients. Based on the women’s comments we revised the questionnaire through several versions until a ninth (pilot) version was formed (Table 1).

We performed pilot testing where women were invited to complete the questionnaire. Four hundred and one consenting women participated in completing the “Questionnaire of the Patient’s Perspective of Bedside Teaching” between October 2018 and June 2019. Research team members distributed to patients attending the antenatal public outpatient department, gynaecology outpatient department, consultant private antenatal clinic, antenatal inpatient ward, postnatal inpatient ward and the gynaecology inpatient ward. Each patient received an explanation as to the reason for the study and what the questionnaire involved. Every patient attending the hospital was eligible for inclusion in the study. Ten women declined to participate. Demographic information is shown in Table 2; rate of answers is shown in Table 3.

**Table 2:** Participants demographics of 401 women who completed the questionnaire

Age (years)	35 (18–70)	
Education	Primary school	4 (1%)
	Secondary school	67 (17%)
	University/third level	330 (82%)
Reason for attendance to hospital	Pregnant	159 (39.65%)
	Postnatal	130 (32.41%)
	Gynaecology	111 (27.68%)
Location of questionnaire completion	Clinic	145 (36%)
	Antenatal Ward	90 (22%)
	Postnatal ward	123 (31%)
	Gynaecology Ward	43 (11%)
Previous participation in teaching	Clinic	Yes 56 (14%)
	Ward	Yes 116 (29%)
	Bedside Teaching	Yes 69 (17%)

**Table 3:** Rate of answers from 401 women who completed the questionnaire. BST = bedside teaching. Items with stars (*) were excluded after statistical analysis

**Question**	**Strongly Disagree**	**Disagree**	**Neutral**	**Agree**	**Strongly Agree**	**Does not apply**	**No Response**
1. Taking part in BST could be an enjoyable experience for me	**10** **(2.5%)**	**24** **(6%)**	**115** **(28.7%)**	**153 (38.2%)**	**82** **(20.4%)**	**13** **(3.2%)**	**4** **(1%)**
2. It would give me satisfaction to feel that I could help in the education of future generations of healthcare professionals by participating in BST	**6** **(1.5%)**	**5** **(1.2%)**	**34** **(8.5%)**	**156 (38.9%)**	**192** **(47.8%)**	**3** **(0.7%)**	**5** **(1.2%)**
3. Medical terms used during BST which I do not understand should be explained to me	**7** **(1.7%)**	**15** **(3.7%)**	**35** **(8.7%)**	**127 (31.7%)**	**202** **(50.4%)**	**10** **(2.5%)**	**5** **(1.2%)**
4. If I felt stressed or was feeling unwell, I should not be asked to participate in BST	**20** **(5%)**	**35** **(8.8%)**	**68** **(17%)**	**134** **(33.4)**	**130** **(32.4%)**	**9** **(2.2%)**	**5** **(1.2%)**
5. By participating in BST, I could possibly learn more about my condition than I had previously known*	**10** **(2.5%)**	**10** **(2.5%)**	**58** **(14.5%)**	**189 (27.1%)**	**118** **(29.4%)**	**11** **(2.7%)**	**5** **(1.2%)**
6. Hearing details about why I am here being discussed in BST may make me feel anxious*	**63 (15.7%)**	**141 (35.2%)**	**91** **(22.7%)**	**69 (17.2%)**	**21** **(5.2%)**	**11** **(2.7%)**	**5** **(1.2%)**
7. I think it is important to check what the students would do with the information they hear about me during BST	**17** **(4.2%)**	**37** **(9.2%)**	**101** **(25.2%)**	**148** **(37%)**	**88** **(22%)**	**5** **(1.2%)**	**5** **(1.2%)**
8. Before participating in BST, the students should get my verbal consent	**9** **(2.2%)**	**18** **(4.5%)**	**26** **(6.5%)**	**146 (36.4%)**	**192** **(48%)**	**5** **(1.2%)**	**5** **(1.2%)**
9. Before participating in BST, the students should get my written consent	**53 (13.2%)**	**119 (29.7%)**	**82** **(20.5%)**	**70 (17.5%)**	**65** **(16.2%)**	**7** **(1.7%)**	**5** **(1.2%)**
10. I am happy to help teach midwives	**2** **(0.5%)**	**6** **(1.5%)**	**20** **(5%)**	**120** **(30%)**	**237** **(59.1%)**	**11** **(2.7%)**	**5** **(1.2%)**
11. I am happy to help teach junior doctors	**2** **(0.5%)**	**5** **(1.2%)**	**2** **1 (5.3%)**	**123 (30.7%)**	**240** **(59.9%)**	**5** **(1.2%)**	**5** **(1.2%)**
12. I am happy to help teach medical students	**1** **(0.2%)**	**7** **(1.7%)**	**23** **(5.7%)**	**127 (31.7%)**	**233** **(58.1%)**	**5** **(1.2%)**	**5** **(1.2%)**
13. It is important to me that the team remains professional throughout BST	**1** **(0.2%)**	**3** **(0.7%)**	**20** **(5%)**	**103 (25.7%)**	**264** **(65.8%)**	**5** **(1.2%)**	**5** **(1.2%)**
14. I feel if asked to participation in BST I would be able to say no*	**10** **(2.5%)**	**22** **(5.5%)**	**34** **(8.5%)**	**163 (40.6%)**	**161** **(40.2%)**	**6** **(1.5%)**	**5** **(1.2%)**
15. I feel proud that women are in a unique position to help educate students about women’s health	**5** **(1.2%)**	**3** **(0.8%)**	**59** **(14.7%)**	**139 (34.7%)**	**186** **(46.4%)**	**4** **(1%)**	**5** **(1.2%)**
16. I am happy to follow the advice of a midwife or doctor when it comes to making a decision for clinical care	**5** **(1.2%)**	**0**	**15** **(3.8%)**	**129 (32.2%)**	**242** **(60.4%)**	**5** **(1.2%)**	**5** **(1.2%)**

For the purposes of reporting in the text, “*strongly disagree*” “*disagree*” and “*neutral*” were amalgamated, as were “*agree*” and “*strongly agree*”. Overall the rate of support for BST was positive, with a clear majority of women reporting that they were happy to teach junior doctors (92.8%), midwives (92.7%) and medical students (92.1%) (*p* = NS). Findings from the qualitative study were reiterated, with a majority of women (82.9%) reiterating a pride that pregnant women are in a “*unique position to help educate students about pregnancy*”. Women agreed that they should be actively included in BST by having medical terms that they were unfamiliar explained to them (85.2%), and that they may actually learn more about their own condition by participation in BST (79.7%).

Teaching points for faculty that were raised by patients during this study, and reiterated in the consensus of results from participants were as follows: the importance of professionalism by the team during BST (83.1%), the wish for either verbal (86.4%) or written (34.7%) consent from the patients prior to participation in BST and providing information on what the personal information obtained during BST would be used for (60.4%). Just under a quarter of women agreed that hearing details of their condition being discussed may distress them (23.4%) reiterating the importance of the facilitator being aware of non-verbal communication and sensitivity.

A clear majority of women believed that they should not even be asked to participate in BST should they feel stressed or unwell (68.2%). Even more women reported that they would be able to say “no” should they not wish to participate (83.1%).

**Fig. 1:**
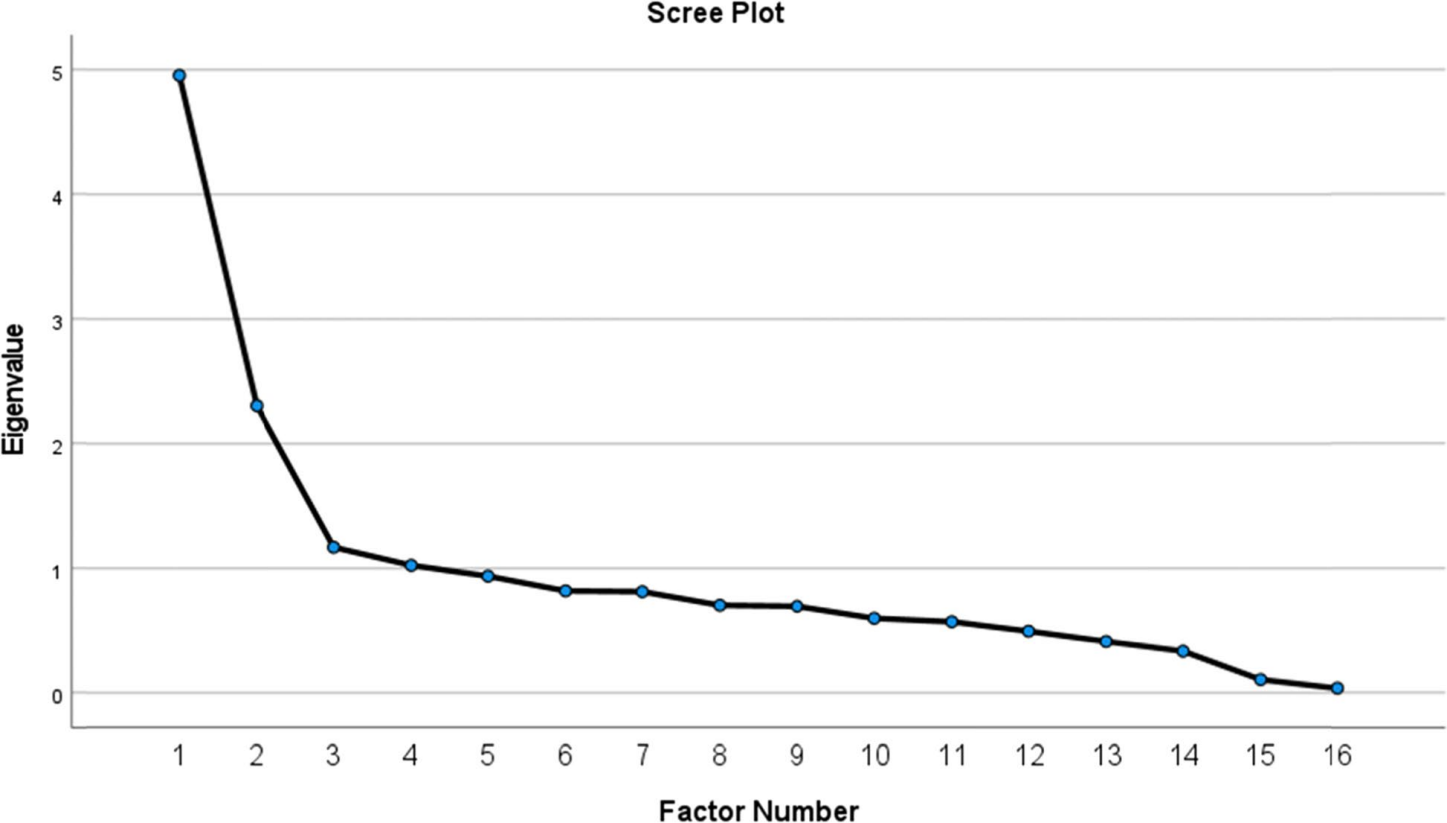
Scree plot of Exploratory Factor Analysis (EFA). The numeric values of the first four Eigan values, with associated % variance explained in parentheses: Factor 1 = 4.96 (30.97%), Factor 2 = 2.31 (14.15%), Factor 3 = 1.17 (7.32%), Factor 4 = 1.03 (6.41%).

Statistical analysis: we conducted an exploratory factor analysis (EFA) in SPSS using maximum likelihood estimation and promax rotation (i.e., allowing the factors to be correlated). The Eigenvalues > 1 suggested up to four factors and Scree plot suggested two factors (Fig. 1). A two-factor solution showed a clear distinction, with three items dropping out with loadings less than 0.3 (#5, 6, 14), and one item (#13) that cross-loading onto both factors. Given the relatively similar factor loadings for this item (0.36 and 0.37), the Cronbach’s alpha with/without was conducted and suggested it should be retained for the second factor. The final two-factor solution explained 45% of the variance among the variables and the factors were not highly correlated (*r* = 0.10); this indicates that the subscales or factors are tapping into distinct constructs. These factors were named as “*Educate Medical Professionals*” (Cronbach’s 0.88) and “*Conditions for Participation*” (Cronbach’s 0.60) (Table 4).

**Table 4:** Loadings for the two-factor solution; *BST: Bedside Teaching. Note:* Factor loadings lower than 0.3 are omitted for clarity (items 5, 6, and 14 in the version for pilot testing)

Loadings for the two-factor solution			
Item #		*Educate Medical Professionals*	*Conditions for Participation*
1	Taking part in BST could be an enjoyable experience for me	0.42	
2	It would give me satisfaction to feel that I could help in the education of future generations of health care professionals by participating in BST	0.62	
10	I am happy to teach midwives	0.98	
11	I am happy to teach junior doctors	0.99	
12	I am happy to teach medical students	0.93	
15	I feel proud that pregnant women are in a unique position to help to educate students about pregnancy during BST	0.53	
16	I am happy to follow the advice of a midwife or doctor when it comes to making a decision for clinical care	0.49	
13	It is important to me that the team remains professional throughout BST		0.37
3	Medical terms used during BST which I do not understand should be explained to me		0.41
4	If I felt stressed or was feeling unwell, I should not be asked to participate in BST		0.37
7	I think it is important to check what the students would do with the information they hear about me during BST		0.57
8	Before participating in BST, the students should get my verbal consent		0.56
9	Before participating in BST, the students should get my written consent		0.50

Next, we performed exploratory analyses aimed to understand the demographic and care setting conditions (e.g., clinic, ward, bedside) which might influence the pattern of response on these subscales. There was no bivariate correlation between age or level of education with either subscale. A series of independent t-tests compared those who had, and had not, participated in each care setting to examine discriminant validity, or the extent to which the scale can help distinguish between potential subgroups in a population. There was no significant difference on either subscale for teaching in the clinic. There was, however, a significant difference for participation in teaching in the ward and bedside; participants who had participated in teaching in the ward (t(386) = -0.19, *p* < 0.01) and bedside (t(386) = -0.19, *p* < 0.05) reported higher desire to *Educate medical professionals* than those that had not. This pattern of findings provides support for discriminant validity; that is, participants who endorsed higher levels of wanting to educate medical professionals also opted into such choices in the more intimate settings of care, the ward and bedside.

## Discussion

This study validated a questionnaire studying women’s opinions of bedside teaching, showing broad support for the importance of BST in medical education and providing some guidance for conditions under which women can participate.

Despite the multiple questionnaires that are used in medical education, there was previously lack of guidance in how to best design a survey [31, 32]. This lack of a validation process can lead to concerns regarding the validity and reliability of the data [30, 32], making it more difficult to draw conclusions from the research or apply it to different situations. A poorly designed study may analyse what the *researcher* considers to be important rather than what is truly important to the subject under study. The publication of an AMEE guide to develop questionnaires has provided a much needed review of this area, and has been cited in the development of questionnaires on undergraduate community based clinical training [33], lifelong learning skills [34], teaching sustainable healthcare education [35], use of educational podcasts [36] and measuring cognitive overload [37]. In a wonderful paper reviewing the “*seven deadly sins*” in educational research, the first author of the AMEE guideline reports the sin of “*ignoring the importance of measurement*” where researchers may naively use a measure that has not been tested that may only serve to confuse results and “*potentially taint the field with contradictory or implausible findings*” [38]. It is the aim of our research group that the methodical following of guidance in developing, validating and applying this questionnaire, and the multidisciplinary work by clinicians, academics, statisticians and psychologists would strengthen the validity and reliability of the results reported.

An important finding of this paper is that there is no correlation between support for BST and level of education. It is crucial that future clinicians have exposure to the full spectrum of patients to learn to provide respectful, non-discriminatory care. It is well recognised that medicine is seen as an “elite” profession, with students more likely to come from wealthy, well-educated families [39], though efforts are ongoing for the student population to be more representative of the general population [40]. Patients involved in medical education must represent the background population. As an example, our previous qualitative study of 23 women who had participated in BST, the majority (73%) had completed tertiary level education [25], compared to 42% in the general population [25, 41]. If university students are taught by university educated lecturers, and are exposed as students mainly to university educated patients then it will be more challenging to provide care to the general population, which is inclusive of non-university educated populations.

Studies exploring women’s attitudes towards medical student teaching often concentrate on student involvement in clinical care [42, 43]. One study specifically investigated the opinions of those who had definitely declined medical student involvement, where the majority (61%) of the 78 women who declined student care cited a desire for privacy. Another study reported that the highest rate of patient refusal across the specialities was within Obstetrics and Gynaecology [19] though this should be taken within the cultural context that the study was written (i.e. the Middle East). It is heartening in our study that the vast majority of patients supported their involvement in BST and were willing to participate. Patient opinions, willingness and criteria to participate and limitations are crucially important factors in medical education. Just as clinical decision making focuses on shared decision making, so too should medical education focus on the patient as an expert in their own disease and in their ability to participate in medical education or not [44, 45]. Exploring patients own opinions and beliefs strengthens shared decision making and empowers the mutual relationship between clinicians, students and patients to everyone’s benefit. Important qualifiers to these results would be that consent may differ in different situations, such as an antenatal clinic compared to a birthing unit or operating theatre, and that theoretical consent does not imply practical involvement.

Given the incredible changes that have occurred in medical education over the last year it seemed initially naïve to write a paper advocating for BST when our world is now one of social distancing and infection control. Similar to other areas of education, our curriculum has pivoted more to an online base, but in this pivoting has come a real realisation by us of the vital importance of patient contact and hospital attendance. The importance of informal learning on placements, the value of the community of practice and the crucial place of the patient in medical education have all become clearer. It is even more important to protect clinical placements for students close to graduation so that they can truly be fit to practice as new doctors. As a result, several initiatives have promoted a “new” BST in an effort to marry the new infection control protocols and guidelines with medical education requirements. Educators have proposed “virtual” teaching rounds with patients diagnosed with COVID-19, allowing students to meet patients diagnosed with the infection and the patients to share their perspectives of their diagnosis [46]. This allows students to be protected but still benefit from the multiple teaching points that are available from this new form of BST. In general medicine, a “*bedside to website*” teaching round allows students to “*telehealth clerk*” a patient, taking a detailed history using secure teleconferencing and requesting a tutor to perform the relevant physical examinations [47]. Students have deemed these rounds to be as good as traditional BST, with the exception of limitations of physical examinations. Proposed strategies to foster bedside teaching [48] can be adapted to the pandemic clinical environment, as the ever “*changing paradigms*” [49] mean that we value conceptual learning strategies of making connections, scaffolding and sharing stories even more. These are times that role modelling humanistic behaviour during BST is even more important, and also facilitates emotionally debriefing after a focused BST which is focused on the needs of the patient [15].

A strength of this study was the systematic validation of the questionnaire following every step recommended within the AMEE guidelines. A limitation was that the study was validated in one patient group (women attending for obstetrics or gynaecology conditions) in one clinical setting (a tertiary level university teaching hospital in the Republic of Ireland). In addition, as this is a teaching hospital with a strong culture of patient focused education, patients may well be biased towards student learning, either choosing to attend as this is a teaching hospital or “buying in” to this culture.

In future research projects we aim to pilot this questionnaire in other patient populations, including men and children as well as other specialities such as General Medicine or Surgery. A future study will also allow further statistical analysis including confirmatory factor analysis (CFA) and structural equation models (SEM). It would be also important to pilot the questionnaire in other clinical environments and countries; this would not involve validation of the questionnaire within these population as a primary step.

## Conclusion

This study has validated a questionnaire on patients opinion of bedside teaching, a corner stone of medical education for generations. With the increase in virtual learning as a result of the COVID-19 pandemic, learning from patients directly and becoming part of a community of practice has become even more crucial for education of students in healthcare, whether medical, nursing, midwifery, physiotherapy, occupational therapy or any other healthcare professional. When making decisions about return to clinical practice after the pandemic, patient opinions are crucial and use of questionnaires can help educators and clinicians gather opinions to allow the patients voice be heard as part of the discussion.

## Data Availability

The datasets generated during and/or analysed during the current study are available from the corresponding author on reasonable request.

## Abbreviations

AMEE: Association for Medical Education in Europe
BST: Bedside Teaching
EFA: Exploratory Factor Analysis
